# Performance characteristics of a modified HIV-1 drug resistance genotyping method for use in resource-limited settings

**DOI:** 10.12688/f1000research.20083.1

**Published:** 2019-08-28

**Authors:** Edwin O. Magomere, Donald D. Nyangahu, Sammy Kimoloi, Brenda A. Webala, Bartholomew N. Ondigo

**Affiliations:** 1 Department of Biochemistry and Molecular Biology, Egerton University, Nakuru, Nakuru, 20115, Kenya; 2 Center for Global Infectious Disease Research, Seattle Children's Research Institute, Seattle, Washington, USA; 3 Department of Pediatrics, University of Washington, Seattle, Seattle, Washington, USA; 4 Department of Medical Laboratory Sciences, Masinde Muliro University of Science and Technology, Kakamega, Kakamega, Kenya; 5 Faculty of Health Sciences, Egerton University, Nakuru, Nakuru, Kenya; 6 Center for Global Health Research, Kenya Medical Research Institute, Kisumu, Kisumu, Kenya; 7 Laboratory of Malaria Immunology and Vaccinology, National Institute of Allergy and Infectious Diseases, NIH, USA, Bethesda, Maryland, USA

**Keywords:** HIV-1 drug resistance testing, Assay validation, Accuracy, Precision, Reproducibility, Amplification sensitivity

## Abstract

**Background:** HIV-1 drug resistance (HIVDR) assays are critical components of HIV clinical management programs in the face of emerging drug resistance. However, the high costs associated with existing commercial HIVDR assays prohibit their routine usage in resource-limited settings. We present the performance characteristics of a modified commercial HIVDR testing assay.

**Methods:** A total of 26 plasma samples were used to validate and assess the accuracy, precision, reproducibility and amplification sensitivity of a modified HIVDR assay by HIV genotyping. In addition, a cost comparison between the original and the modified assay was performed using the ingredient costing approach.

**Results:** The performance characteristics of the modified assay were in agreement with the original assay. Accuracy, precision and reproducibility showed nucleotide sequence identity of 98.5% (confidence interval (CI), 97.9–99.1%), 98.67% (CI, 98.1–99.23) and 98.7% (CI, 98.1–99.3), respectively.  There was no difference in the type of mutations detected by the two assays (χ ^2 ^= 2.36, *p* = 0.26). Precision and reproducibility showed significant mutation agreement between replicates (kappa = 0.79 and 0.78), respectively ( *p *< 0.05). The amplification sensitivity of the modified assay was 100% and 62.5% for viremia ≥1000 copies/ml and <1000 copies/ml respectively. Our assay modification translates to a 39.2% reduction in the cost of reagents.

**Conclusions:** Our findings underscore the potential of modifying commercially available HIVDR testing assays into cost-effective, yet accurate assays for use in resource-limited settings.

## Introduction

There has been an unprecedented increase in support for HIV diagnosis, treatment and monitoring programmes. Various multinational groups including the U.S. President’s Emergency Plan for AIDS Relief (PEPFAR) and Global Fund have increased funding of HIV management programs leading to improved access to antiretroviral therapy (ART) ^1, 2^. The outcome of the expanded access to ART is significant decline of HIV/AIDS-associated morbidity and mortality ^2^. As a result, there has been an increase in both life expectancy and duration for patients on lifelong ART translating to high risk of HIV drug resistance (HIVDR) development. To sustain the success achieved following improved ART coverage, continuous clinical, virologic and immunological monitoring of patients on ART is required to ensure these positive treatment outcomes are maintained ^3, 4^. Unfortunately, development and transmission of HIV drug resistance threatens to derail these achievements ^5^. HIV drug resistance testing (DRT) is routinely used for clinical care in high-income countries; however, it is not available to a majority of patients in resource-limited settings due to the high costs of implementation and limited trained manpower.

The World Health Organization (WHO) launched the Global Action Plan (GAP) against HIVDR for resource-limited countries whereby two of the proposed approaches included continuous innovation and capacity building of laboratory staff ^6^. Scientists have attempted to develop alternative affordable methods for HIV drug resistance monitoring to improve access ^5, 7– 9^. One such test was developed by Centre for Disease Control and Prevention (CDC) and is currently distributed by Thermo Fisher Scientific ^10^, and is herein referred to as the original assay. The assay is used to genotype the genetically diverse HIV-1 virus from plasma samples to detect resistance mutations in the protease and reverse transcriptase genes with a good subtype inclusivity rate compared to other commercial DRT assays in the market ^11^.

In the present study, we modified this commercial HIV DRT protocol by a 50% reduction of reagent volumes for nested PCR and cycle sequencing reactions for genotyping of HIV-1 drug resistance. The performance characteristics of the modified assay were assessed and evaluated for suitability and reliability using WHO guidelines for assay validation. In addition, cost comparison was performed to determine the cost implication of the assay modification.

## Methods

### Samples

A total of 26 blood samples were collected in EDTA tubes from patients attending Kenyatta National Hospital HIV clinic laboratory for routine viral load monitoring. Plasma was separated by centrifugation at 2000 *g* for 10 minutes. The plasma samples were stored at -80°C freezer (Nuve, Ankara, Turkey). Remnant plasma samples were used for this study.

### Ethical approval

Ethical clearance for the study was obtained from University of Nairobi-Kenyatta National Hospital Ethics Review Committee (UoN-KNH-ERC). The need for consent was waived since remnant plasma from HIV viral load testing were used in this study.

### Viral RNA extraction

HIV-1 RNA was extracted from 500 µl plasma using the PureLink ^TM^ extraction kit according to manufacturer’s instructions (Invitrogen, Carlsbad, CA, USA). Briefly, 25 μl proteinase K and 500 μl plasma were transferred into 2-ml microcentrifuge tube. Lysis buffer (500 μl) was added to the mixture, vortexed for 15 seconds, and incubated at 56°C for 15 minutes. Next, 500 μl of 96% ethanol was added to the reaction tube, and vortexed for 15 seconds and incubated at room temperature for 5 minutes. The lysate was transferred to sterile viral spin column, washed twice with 500 μl of wash buffer and finally eluted in 40 μl of elution buffer. RNA was stored at -80°C. The extraction procedure was similar for both the original and modified method.

### HIV DRT

***Original genotyping method.*** The Thermo Fisher Scientific ^TM^ HIV Genotyping workflow that amplifies a 1.1-kb fragment covering codons 6–99 of the protease gene and codons 1–251 of the reverse transcriptase (RT) gene was implemented according to manufacturer’s instructions ^10^. Briefly, reverse transcriptase PCR and nested PCR were performed using the HIV-1 Genotyping Kit: Amplification Module. Primers used are shown in Table 1. Cycle sequencing was performed using the HIV-1 Genotyping Kit: Cycle Sequencing Module. The resulting cycle sequencing products were analyzed using an ABI 3730 genetic analyzer. The consensus sequences were generated using ReCall (requires free registration) and drug resistance mutations were interpreted using the Stanford HIVdb genotyping resistance interpretation algorithm.

**Table 1.** Primers used in the RT-PCR and nested PCR steps.

	Primer Sequence (5’–3’)	Step	Fragment size
1	F1a, 5’-TGAARGAITGYACTGARAGRCAGGCTAAT	RT-PCR	2057 to 2085
2	F1b, 5’-ACTGARAGRCAGGCTAATTTTTTAG	RT-PCR	2068 to 2092
3	R, 5’-ATCCCTGCATAAATCTGACTTGC	RT-PCR	3370 to 3348
4	PRT-F2 ,5’-CTTTARCTTCCCTCARATCACTCT	NESTED-PCR	2243 to 2266)
5	RT-R2, 5’-CTTCTGTATGTCATTGACAGTCC	NESTED-PCR	3326 to 3304

***Modified genotyping system*** Specific reaction steps of the original protocol were modified by reducing the reagent volumes by half.

*The HIV Genotyping kit:* Amplification module was used for RT-PCR according to the manufacturer’s instructions. Briefly, 10 µl RNA was denatured by incubating at 65°C for 10 minutes and added to 40 µl RT-PCR Master Mix containing SuperScript ^TM^ III One-Step RT-PCR with Platinum ^TM^Taq High Fidelity Enzyme. The reaction conditions for RT-PCR were 50°C for 45 minutes where first-strand cDNA synthesis was performed. Enzyme inactivation and denaturation of cDNA-RNA hybrid was accomplished by incubating the reaction at 94°C for 2 minutes. Second-strand synthesis and PCR amplification was carried out in 40 cycles of 94°C for 15 seconds, 50°C for 20 seconds, 72°C for 2 minutes and a final extension for 10 minutes at 72°C using Veriti thermocycler.

Nested PCR reaction mix was prepared by adding 2 μl RT-PCR product to 23.7 μl of nested mastermix containing 0.12 mM of each of inner primers, 1X GeneAmp Gold Buffer II, 2 mM MgCl _2_, 400 mM each dNTP and 0.25 μl AmpliTaq Gold™ LD DNA Polymerase enzyme (Applied Biosystem, CA, USA). The target pol region was amplified using Veriti Thermocycler in a final reaction volume of 25.7 µl (Applied Biosystems, CA, USA). The reaction conditions were 94 ^0^C for 4 minutes, 40 cycles of 94°C for 15 seconds, 55°C for 20 seconds, 72°C for 2 minutes and a final extension at 72°C for 10 minutes. Nested PCR products of 1.1 kb were confirmed by gel electrophoresis. A 5 μl aliquot of nested PCR product was added to 4 μl ExoSAP-IT ^TM^ PCR Purification Reagent) and incubated at 37°C for 15 min and 80°C for 15 minutes in a Veriti thermocycler.

Cycle sequencing was performed using the HIV Genotyping kit: Cycle Sequencing module. Six overlapping primers, labelled F1, F2, F3, R1, R2, and R3 were used. One microliter nested PCR product was added to 9 µl of each cycle sequencing mix. The cycle sequencing reaction conditions were 25 cycles of 96°C for 10 seconds, 50°C for 5 seconds, and 60°C for 4 minutes.

The Big Dye XTerminator purification kit was used to purify the sequencing reaction by adding 10 µl of the Big Dye XTerminator and 45 µl SAM solution to cycle sequencing products. The reaction plate was vortexed at 1,800 rpm for 30 minutes. The plate was then centrifuged at 1000 *g* for 2 minutes at room temperature. Next, 30 μl of purified cycle sequencing products were transferred to a reaction plate and analyzed using an ABI 3730 genetic analyzer (Applied Biosystem, CA, USA). Sequences were generated using ReCall and drug resistance mutations interpreted using the Stanford HIVdb genotyping resistance interpretation algorithm (output files available as *Extended data* ^12^) and the International AIDS Society (IAS) 2011 mutation list ^13^.

### Modified assay validation

Performance characteristics of the modified assay were assessed using the WHO/HIV ResNet guidelines, including accuracy, precision, reproducibility and amplification sensitivity ^14^.

***Accuracy.*** Accuracy refers to the agreement between a result and an expected reference value. In genotyping assays, nucleotide sequence identity is used. Ten samples were analyzed using both methods and the degree of concordance in mutations identified was compared based on the 2017 IAS mutations list ^13^. Nucleotide sequence identity between the paired sequences was assessed using the EMBOSS pairwise alignment tool. The WHO recommends 90% nucleotide sequence similarity as the cutoff point for assay performance characteristics ^14^.

***Precision.*** This is the ability of an assay to generate the same result on multiple replicates of the same sample within a test run. Three samples were analyzed using the modified method in *n*=4 replicates. The degree of concordance of detected mutations within replicates was determined. Nucleotide sequence identity was also determined using the EMBOSS program for pairwise alignment ^14^.

***Reproducibility.*** This is the ability of a test to produce the same result on multiple aliquots of the same sample in different test runs. Ten samples were analyzed in duplicates using the modified method on different days. Nucleotide sequence identity of sequences obtained was assessed using EMBOSS program for pairwise alignment ^14^.

***Amplification sensitivity.*** Amplification sensitivity is defined as the percentage of successful genotyping tests amongst specimens with a specific viral load range. In this study, sixteen samples with viral loads ranging between 207 and 86,040 copies/ml were analyzed using the modified assay to determine the viral load ranges at which ≥95% of the samples were successfully genotyped ^14^.

### Reagent cost comparison

An ingredient costing approach was utilized to estimate reagent costs for both original and modified assays at RNA extraction, DNA/RNA amplification, gel electrophoresis, and sequencing steps. All costs were converted to US dollar using 2019 conversion rate.

### Statistical analyses

Quantitative variables were expressed as mean ± standard deviations (SD). The McNemar test was used to assess significance in the discordant mutations between the modified and the original assay. Precision and reproducibility were assessed using the Cohen kappa statistic. Wilcoxon signed-rank test was used to compare the original and modified assays in base calling for mixed bases between the two methods. Statistical Package for Social Sciences (SPSS) version 22 (IBM, NY, USA) was used for all data analysis.

## Results

### Validation of the modified HIV-1 drug resistance genotyping assay

***Accuracy of sequence identity and mutation detection.*** The mean nucleotide identity was 98.5% (confidence interval (CI), 97.92–99.1%). A total of 68 mutations were detected, including 9 (13%) mutations in the protease gene and 59 (87%) in the reverse transcriptase (RT) gene as shown in Table 2. The original assay detected 67 drug resistance mutations, including 2 mutations (V106I and K225H) which were missed by the modified assay. On the other hand, the modified assay detected 68 drug resistance mutations, including four mutations (D67DG, K101KPQT, K70KN and I50IL) which were not detected by the original method. A total of six mutations were discordant (V106I, P225H, D67DN, I50IL, K101KPQT, and K70KN). Of the six discordant drug resistance mutations, two were completely discordant (V106I and P225H) while four (D67DN, K101KPQT, K70KN and I50IL) were partially discordant. Three of the discordant mutations (D67DN, K101KPQT, and K70KN) were detected as mixtures in the modified assay as non-mixtures (D67N, K101P and K70N) in the original assay and as shown in Table 3. A high mutation concordance was obtained between the two assays at χ ^2^ = 2.36, *p =* 0.26. The significance of mixture base-calling between the two methods was determined using the Wilcoxon signed rank test, which showed no significant difference in mixtures detected by the two methods at *p* = 0.089.

**Table 2.** Summary of the paired sequence comparisons performed by the original and the modified assay.

Basis of comparison	Original vs. Modified
Number of samples	10 vs. 10
Nucleotide identity	98.5% (CI, 97.9 – 99.1%)
Number of mutations detected	67 vs. 68
Number of discordant mutations	6 (2 ^**ᵃ**^ vs. 4 ^**ᵇ**^)

**ᵃ**Discordant mutations detected by the original assay. **ᵇ**Discordant mutations detected by the modified assay.

**Table 3.** Pairwise comparison of drug resistance mutations identified by the original assay with those identified by the modified assay.

ID	Reverse transcriptase gene		Protease gene	
	Original	Modified	Original	Modified
1	M184V, H221Y	M41L, M184V, H221Y		
2				
3	V106VM, V179E, Y181C, Y188YC, A62V, V75I, K219KE	A62V, V75I, D67DN ^**ᵇ**^, K219KE, V106VM, V179E, Y181C, Y188YC		I50IL ^**ᵇ**^
4	M41L, M184V, L210W, T215FY, A98G, V106I ^**ᵃ**^, Y188L	M184V, L210W, T215FY, Y188L, K238KT	M46I, I84V	M46MI, I84V
5	M184V, K103N, V108I, P225H ^**ᵃ**^	M184V, K103N, V108I		
6	K65R, Y115F, M184V, V106M, G190A	K65R, Y115F, M184V, V106M, G190A		
7	D67N, K70R, L74I, M184V, K219Q, A98G, K103N, V108I, E138Q, V179L, K238T, L74V, Y115F, M184V	D67N, K70R, L74I, M184V, K219Q, A98G, K103N, V108I, E138Q, V179L, K238T, L74V		
8	K219KQ, Y181C, Y188L, H221Y, M41L	L74V, Y115F, M184V, K219Q, Y181C, Y188L, H221Y		
9	D67N, K70R, M184V, T215F, K219E, A98G, V108VI, Y181C, Y181YC	D67N, T215F, K219E, A98G, K101KPQT ^**ᵇ**^, E138Q, Y181YC, K238N	M46I, I54V, V82A	M46I, I54IV, V82A
10	D67DG, K70N, M184V, K101P, E138Q	D67DG, K70KN ^**ᵇ**^, M184V, T215F, K219E, A98G, K101P, Y181YC	M46MI, I54IV, V82VA	M46MI, I54IV, V82VA

**ᵃ**Discordant drug resistance associated mutations detected in original assay. **ᵇ**Discordant drug resistance associated mutations detected in the modified assay.

***Precision and reproducibility of the modified assay.*** Assessment to examine the precision (intra-assay precision) and reproducibility (inter-assay precision) of the modified assay were performed by analyzing *n*=3 samples in quadruplicate in a single test run for precision. The mean nucleotide sequence identity within the replicates was 98.67% (CI, 98.1–99.3). Reproducibility was assessed by testing 10 samples in duplicate on different days using the modified assay. The mean nucleotide sequence identity was 98.6% (CI, 98.2–99.0). The overall agreement of drug resistance mutations detected by precision and reproducibility was significant at kappa value of 0.792 and 0.778, respectively, as shown in Table 4.

**Table 4.** Precision and reproducibility of genotyping using the original and modified assay.

Parameter	Number of samples	Replicates	kappa value	*P* value	Nucleotide identity
Precision	3	12	0.792	0.58	98.67%
Reproducibility	10	20	0.778	0.4	98.6%

***Amplification sensitivity of the modified assay.*** A total of 16 samples with a median viral load of 832.5 copies/ml (IQR 403.5–2454.25) were used to assess amplification sensitivity. The modified assay showed an amplification sensitivity of 100% for samples with viral load ≥1000 copies/ml and 62.5% for samples with viral load <1000 copies/ml as shown in Table 5.

**Table 5.** Viral load levels and HIV-1 subtypes detected in plasma samples used for amplification sensitivity testing.

Sample ID	VL (Copies/ml)	Subtype
1	86,040	A1
2	12,155	A1
3	2,802	D
4	2,700	D
5	1,717	A1
6	1,547	A1
7	1,100	C
8	1,040	A1
9	625	D
10 ^ᵇ^	612	
11	514	C
12 ^ᵇ^	459	
13	385	A1
14	312	A1
15	214	A1
16 ^ᵇ^	207	

ᵇ Failed to amplify

### Comparison of costs of reagents between the original and modified assays

After a 50% reduction in reagent volumes in the amplification and sequencing reactions, we compared the cost between the original and the modified method. The cost of analyzing one sample from extraction to sequencing was decreased from $97 to $59. This represents about 39.2% cost reduction as shown in Table 6.

**Table 6.** Cost comparison at several steps of the procedure of HIV drug resistance testing.

Reaction steps	Original assay, $	Modified assay, $
RNA extraction	4.2	4.2
DNA/RNA amplification	37.2	19.2
Gel electrophoresis	1.2	1.2
Sequencing	54.4	34.4
**Total cost**	**97**	**59**

## Discussion

Here, we show that the results of the modified assay are comparable to those produced by the original assay in the performance characteristics analyzed including accuracy, precision, reproducibility and amplification sensitivity. We observed a high concordance of drug resistance mutations detected by both original and modified assays. When testing for accuracy, we observed high nucleotide sequence similarity (98.5±0.94%) versus original assay. In addition, we reported 98.67% (CI, 98.1–99.3) sequence similarity when assessing precision and 98.6% (CI, 98.2–99.0) when assessing reproducibility of the modified assay ^15^. Furthermore, the modified assay showed 100% sensitivity for VL >1000 copies/ml which is the recommended indicator for HIV treatment failure ^16^. In general, the modified assay met all the requirements in the WHO Genotyping assay validation recommendations. Altogether our findings demonstrate that reducing the volumes of reagents in the original assay by up to half results in a low-cost HIV drug resistance assay that could be utilized in resource-limited settings without compromising the quality of testing.

Accuracy was assessed by analyzing 10 samples with viral load ranges (4258 –78924 copies per ml) by both the original and the modified assays and all 10 samples were successfully amplified and genotyped with high nucleotide sequence identity (98.5±0.94%). All the clinically relevant mutations were reproducible by both methods in spite of some minor discordance between the two methods. The WHO recommends a minimum of 90% sequence similarity for genotyping assays ^14^. Our findings of 98.5±0.94% nucleotide sequence similarity are in agreement with those reported by Zhou *et al*. ^7^ who reported 99.41%. In addition, analysis of mutation concordance between the original and the modified assay showed high mutation concordance also reported by Inzaule *et al*. ^8^. Our findings for mutation concordance (93%) were lower than reported by Manasa *et al*. (100%) ^5^. The modified assay precision assessment showed a high degree of nucleotide sequence identity within replicates. Similar results for precision (98.22%) were reported by Zhou *et al*. ^7^. High sequence similarity was also obtained for reproducibility of the modified assay. Our findings for reproducibility (98.6%) were similar to those reported by Zhou *et al*., who obtained (98.94%) ^7^. Amplification sensitivity was defined as the percentage of samples successfully genotyped at a given plasma viral load range. In this study, all samples with viral load ≥1000 copies/ml were successfully genotyped, a finding that was similar to that reported by a previous study ^8^. For samples with plasma viral loads <1000 copies/ml, our modified assay successfully genotyped 62.5% of the samples which is slightly below 63.6% reported by a previous study ^8^. The WHO criteria for HIVDR testing requires that patients with persistent viral load ≥1000 copies/ml to be tested for HIV drug resistance ^17^. Our assay was 100% efficient in genotyping these samples. Furthermore, with 62.5% amplification sensitivity for low level viremia (LLV) samples, highlighted the possibility of using the modified assay in genotyping patients with LLV. However, there is need to perform further large-scale studies to clearly establish the performance of the modified method in LLV patients.

We detected six discordant mutations in both original and modified assays. Out of the six discordant mutations, four were mixed-base mutations, which were detected exclusively by the modified assay. The differential detection of discordant mutations between the two assays could be attributed to detection of nucleotide mixtures as a result of subjectivity in basecalling or amplification bias ^18, 19^. Previous studies have also reported a significant contribution of nucleotide mixtures to discrepancies of mutations between Viroseq and an in-house assay ^6, 7^.

The comparison of cost between the two assays revealed a reduction of HIV drug resistance testing cost by 39.2% per test. These findings suggest that access to HIV drug resistance services in resource-limited settings could be increased through innovative modifications of existing commercially available assays. A small sample size was used because this was a small modification of a validated test already in use. The applicability of this assay can be demonstrated further by testing a larger number of samples. One limitation of the study is the samples used were from patients failing first line treatment and not exposed to protease inhibitors leading to under representation of drug resistant mutations in the protease gene.

## Conclusion

Our findings underscore the potential utility of a modified cost-effective HIV-1 drug resistance assay for testing plasma samples in resource-limited settings. The performance characteristics of the modified assay were satisfactory and therefore the cheaper assay could be one of the approaches of increasing access to HIVDR tests.

## Data availability

### Underlying data

GenBank accession numbers for HIV-1 pol and gag sequences isolated from each participant using each technique are available in Table 7.

**Table 7.** Accession numbers of HIV-1 sequences generated in this study.

HIV-1 sequence isolate	GenBank accession number	Assay type
19BI03	MN240769	Original
19GB05	MN240770	Original
19KE01	MN240771	Original
19KE02	MN240772	Original
19KE06	MN240773	Original
19KE07	MN240774	Original
19KE08	MN240775	Original
19KE09	MN240776	Original
19KE10	MN240777	Original
19UG04	MN240778	Original
BI03	MN240779	Modified
GB05	MN240780	Modified
KE01	MN240781	Modified
KE012	MN240782	Precision run
KE014	MN240783	Precision run
KE02	MN240784	Modified
KE06	MN240785	Modified
KE07	MN240786	Modified
KE08	MN240787	Modified
KE09	MN240788	Modified
KE10	MN240789	Modified
SE011	MN240790	Modified
UG013	MN240791	Precision run
UG04	MN240792	Modified

### Extended data

Figshare: Performance Characteristics of a Modified HIV-1 Drug Resistance Genotyping Method for use in Resource Limited Settings: https://doi.org/10.6084/m9.figshare.9114833 ^12^.

This study contains the following extended data:

- Accuracy testing data (Stanford HIV Drug Resistance reports; PDF)
- Precision testing data (Stanford HIV Drug Resistance reports; PDF)
- Reproducibility testing data (Stanford HIV Drug Resistance reports; PDF)
- EM_Accuracy_Precision_Repro_AmpSensitivity (summary of HIV Drug Resistance Mutations detected; xlsx)
- Gel images (TIF)

Data are available under the terms of the Creative Commons Attribution 4.0 International license (CC-BY 4.0).
