# Supplementary material for: Performance characteristics of a modified HIV-1 drug resistance genotyping method for use in resource-limited settings
Source: F1000Res. 2019 Aug 28;8:1518. [Version 1] doi: 10.12688/f1000research.20083.1 (PMC6798314; doi:10.12688/f1000research.20083.1)
Supplement: Peer review history

# Peer review: Reviewer response for version 1

**DOI:** 10.5256/f1000research.22050.r53071

**Published:** 2019-10-16

**Authors:** Frank Onyambu

**Affiliations:** 1 University of Nairobi, Nairobi, Kenya; 2 Meru University of Science & Technology, Meru, Kenya

This is an innovative approach to reducing costs of offering HIV drug resistance testing in resource-limited settings. The authors have shown a thorough understanding of the literature in HIV drug resistance testing and implementation in resource-limited settings. They have related their findings to the majority of publications that presenting performance characteristics of in-house HIV drug resistance  tests. It would be interesting for the authors to compare the performance of the ‘modified assay’ with the Viroseq assay as well.

 The authors followed the World Health Organization (WHO) recommendations for validating 'in-house' HIV drug resistance assays and clearly showed that the results of the modified assay were comparable to the commercial assay. Furthermore, the authors have demonstrated that these assay modifications resulted in a significant decrease in cost of implementation. I however wonder why the authors did not present results for reducing reaction volumes for the RT-PCR step. It is conceivable that they may have at least attempted and therefore should comment if the results did not meet the WHO criterion for assay validation.

I confirm that I have read this submission and believe that I have an appropriate level of expertise to confirm that it is of an acceptable scientific standard.

# Peer review: Reviewer response for version 1

**DOI:** 10.5256/f1000research.22050.r53075

**Published:** 2019-09-18

**Authors:** Michael Walekhwa

**Affiliations:** 1 Department of Biomedical Sciences, Kabarak University, Nakuru, Kenya

**Title**

1. Ok.

**Abstract**

1. ‘We present the performance characteristics of a modified commercial HIVDR testing assay.’ Should be changed to ‘this study established the performance characteristics of a modified commercial HIVDR testing assay’.
2. The abstract lacks a recommendation.
3. The key word ‘assay validation’ is missing in the abstract.

**Introduction**

1. ‘Various multinational groups’ use ‘agencies’ instead of ‘groups’.
2. ‘….leading to improved access to antiretroviral therapy (ART)’ replace with ‘leading to improved access to HIV clinical care’. (HIV care is not limited to antiretroviral therapy only).
3. ‘….the outcome of the expanded access to ART is significant decline of HIV/AIDS associated morbidity and mortality.’ Replace with ‘…the outcome of the expanded access to clinical care has been a significant drop in HIV/AIDS related morbidity and mortality.’
4. ‘As a result, there has been an increase in both life expectancy and duration for patients on lifelong ART translating to high risk of HIV drug resistance (HIVDR) development’. This statement has not been brought out clearly & what aspect of ‘duration’ is being implied in this case?
5. ‘……transmission of HIV drug resistance’ add ‘strains’ after resistance.
6. ‘One such test was developed by Centre for Disease Control and Prevention (CDC) and is currently distributed by Thermo Fisher Scientific10, and is herein referred to as the original assay’. Change to ‘One such test, herein referred to as the original assay, was developed by the Centre for Disease Control and Prevention (CDC) and is currently distributed by Thermo Fisher Scientific.’

**Methods**

**Samples**

1. Are 26 samples enough to validate an assay? And how did you arrive upon this sample size?
2. How were the samples collected? By venipuncture or cardiac puncture? Be very clear by which method.
3. After clinic, delete the word ‘laboratory’. Patients attend clinics, not clinic laboratories.
-80 is already a freezing condition. Delete the word ‘freezer’, the statement will still make sense without the subtle repetition.
5. What are ‘remnant plasma samples’? Were they the ones stored in the freezer? What defines them as ‘remnant’?

**Ethical approval **

1. It is claimed here that consent was waived, yet in the sample collection, it is clearly stated that the samples were collected from patients. How and why was the consent waived, and by whom?

**Viral RNA extraction**

1.  Add ‘the’ before manufacturer’s.
2. Microcentrifuge ‘tubes’. Plural.

**HIV DRT**

1. As this was a study validating an assay that relied heavily on reagent volumes, why aren’t the volumes of the original assay and their reaction temperatures not included here?

**Reagent cost comparison**

1. As the year is not out yet and conversion rates keep fluctuating, which conversion rate did you use?

**Provide references for definition of the following words:** Precision, Reproducibility, Accuracy and amplification sensitivity

**Statistics**

1. Significance was set at what *p* value?

**Results**

1. Only two drug resistance genes are targeted in this assay, what informed the decision to arrive on these two sets of genes?
2. Table 5 should be the one introducing the results subtopic. Introduce the samples, the viral loads before the validation assay results are presented.
3. Remove the grids from the tables.

**Discussion**

1.  Use third person singular when discussing your results.
2. You don’t ‘show’ in discussion what has been shown in the results section.
3. Delete ‘altogether’ in the first paragraph.
4. What did Zhou *et al.* ^1^ find out, and which procedure were they modifying? Include the year of the study too. As it has been used as the main reference, is it the only study that supports your findings? Are there studies that contradict your findings, and if there are, why aren’t they included here?
5. If the samples were obtained from patients who had not been exposed to protease inhibitors, why did your study focus on protease inhibitors?

I confirm that I have read this submission and believe that I have an appropriate level of expertise to confirm that it is of an acceptable scientific standard.

## References

[1] : Optimization of a low cost and broadly sensitive genotyping assay for HIV-1 drug resistance surveillance and monitoring in resource-limited settings. *PLoS One*.2011;6(11) : 10.1371/journal.pone.0028184 e28184 10.1371/journal.pone.0028184 22132237PMC3223235
